# Effectiveness of opportunistic osteoporosis screening on chest CT using the DCNN model

**DOI:** 10.1186/s12891-024-07297-1

**Published:** 2024-02-27

**Authors:** Jing Pan, Peng-cheng Lin, Shen-chu Gong, Ze Wang, Rui Cao, Yuan Lv, Kun Zhang, Lin Wang

**Affiliations:** 1 Department of Radiology, Nanjing Hospital of Chinese Medicine Affiliated to Nanjing University of Chinese Medicine, Nanjing, Jiangsu 210000 China; 2 School of Electrical Engineering, Nantong University, Nantong, Jiangsu 226001 China; 3 Department of Radiology, The First People’s Hospital of Nantong/The Second Affiliated Hospital of Nantong University, Nantong, Jiangsu 226001 China

**Keywords:** Osteoporosis, Quantitative CT, Deep learning, Bone mineral density, Chest CT

## Abstract

**Objective:**

To develop and evaluate a deep learning model based on chest CT that achieves favorable performance on opportunistic osteoporosis screening using the lumbar 1 + lumbar 2 vertebral bodies fusion feature images, and explore the feasibility and effectiveness of the model based on the lumbar 1 vertebral body alone.

**Materials and methods:**

The chest CT images of 1048 health check subjects from January 2021 to June were retrospectively collected as the internal dataset (the segmentation model: 548 for training, 100 for tuning and 400 for test. The classification model: 530 for training, 100 for validation and 418 for test set). The subjects were divided into three categories according to the quantitative CT measurements, namely, normal, osteopenia and osteoporosis. First, a deep learning-based segmentation model was constructed, and the dice similarity coefficient(DSC) was used to compare the consistency between the model and manual labelling. Then, two classification models were established, namely, (i) model 1 (fusion feature construction of lumbar vertebral bodies 1 and 2) and (ii) model 2 (feature construction of lumbar 1 alone). Receiver operating characteristic curves were used to evaluate the diagnostic efficacy of the models, and the Delong test was used to compare the areas under the curve.

**Results:**

When the number of images in the training set was 300, the DSC value was 0.951 ± 0.030 in the test set. The results showed that the model 1 diagnosing normal, osteopenia and osteoporosis achieved an AUC of 0.990, 0.952 and 0.980; the model 2 diagnosing normal, osteopenia and osteoporosis achieved an AUC of 0.983, 0.940 and 0.978. The Delong test showed that there was no significant difference in area under the curve (AUC) values between the osteopenia group and osteoporosis group (*P* = 0.210, 0.546), while the AUC value of normal model 2 was higher than that of model 1 (0.990 vs. 0.983, *P* = 0.033).

**Conclusion:**

This study proposed a chest CT deep learning model that achieves favorable performance on opportunistic osteoporosis screening using the lumbar 1 + lumbar 2 vertebral bodies fusion feature images. We further constructed the comparable model based on the lumbar 1 vertebra alone which can shorten the scan length, reduce the radiation dose received by patients, and reduce the training cost of technologists.

## Introduction

Osteoporosis is a metabolic bone disease characterized by systemic or local bone loss and an increased risk of fracture [1]. Given the ageing of the large global population, it is considered a major illness. Osteoporosis is estimated to affect 13.5% of men and 29.0% of women aged 50 and over in China [2]. Worldwide, 19.7% of men and 40.4% of women aged 50 and over suffer from osteoporosis and osteopenia, respectively [3]. Early diagnosis and treatment of osteoporosis can effectively slow the development of bone resorption and reduce the risk of fragility fracture and the incidence of osteoporosis-related complications, alleviating the degree of social burden [4]. The SCOOP(Screening for prevention of fractures in older women) study of fragility fracture prevention screening in older women in the UK confirmed a significant 33% reduction in hip fracture incidence in the intervention group compared with the control group [5]. Therefore, early screening and monitoring are essential for the timely prevention and treatment of osteoporosis [1].

For the diagnosis and screening of osteoporosis, the measurement of vertebral bone mineral density(BMD) is an important indicator recommended by the World Health Organization [6].Currently, Dual-energy X-ray absorptiometry (DXA) is often used as a reference standard for bone mineral density classification to diagnose osteoporosis. But DXA is a two-dimensional imaging technology [7] that is susceptible to scoliosis, facet joint degeneration, soft tissue calcification, especially abdominal aortic calcification and other factors, reducing the accuracy of bone mineral density measurement. Quantitative computed tomography(QCT) is a three-dimensional imaging technology that can quantitatively evaluate vertebral cancellous bone mineral density, which is more sensitive to osteoporosis, and the measurement results are more stable than DXA [8, 9]. However, early screening of osteoporosis is difficult. Miller PD [10] investigated that nearly a quarter of women with high risk factors for osteoporosis have never undergone BMD measurements due to insufficient understanding of fragility fractures, the need for auxiliary hardware equipment to measure BMD, and additional manual operation costs.

In addition to bone mineral density, the geometric features, bone microstructure and density structure changes can also reflect the degree of osteoporosis to a certain extent [11, 12], which manifests as changes in local image characteristics in CT images. There has been a growing interest and utilization of artificial intelligence, specifically deep learning and machine learning, in the field of medical imaging in recent years. A Convolutional neural network(CNN) [13] is a representation learning method that improves the accuracy of osteoporosis diagnosis by constructing a multi-hidden layer model and using a large amount of training set data to identify, extract and learn effective features, including bone mineral density and bone microstructure in such images. In a study based on lumbar CT images of 808 postmenopausal women, Zhang [14] et al. used DXA as the bone mass classification standard to construct a DCNN model for the diagnosis of osteoporosis, with a sensitivity of 68.4%, a specificity of 67.8%, and an AUC of 0.726. Fang [15] et al. used a DenseNet-121 convolutional neural network to accurately identify bone mineral density status(osteoporosis, osteopenia, normal) in lumbar CT images. Jang M [16] et al. used 13,026 chest radiographs and DXA to train a OsPor-screen model. Osteoporosis screening with OsPor-screen model achieved an AUC of 0.91 [95% confidence interval(CI): 0.90–0.92]. Mao L [17] et al. proposed to construct a convolutional neural network model for screening primary osteopenia and osteoporosis based on the lumbar radiographs. The models with images alone achieved moderate sensitivity in classifying osteopenia, in which the highest AUC achieved 0.785. These results suggest that deep learning network could have the potential to be used opportunistic automated screening of patients with osteoporosis in clinical settings. Therefore we plan to draw on the classical encoding and decoding structures and then optimize the model based on these traditional model.

In this study, we aimed to develop and evaluate a chest CT deep learning model that achieves favorable performance on opportunistic osteoporosis screening using the lumbar 1 + lumbar 2 vertebral bodies fusion feature images, and explore whether the model based on the lumbar 1 vertebra alone achieves comparable performance. The establishment of this model can help clinical screening of large sample population, find out the cases that really need clinical intervention, and can also be used for diagnosis and treatment evaluation and follow-up work.

## Materials and methods

This study was a retrospective study. It was approved by the Ethics Committee of Nantong First People’s Hospital (No.2021KT028), who waived the need for informed consent. The study protocol was implemented according to the Good Clinical Practice guidelines defined by the Helsinki Declaration and the International Conference on Harmonization (ICH).

### Study design and patient population

In this study, all images were from Ingenuity Core 128 CT (Philips Health Care, Holland). Data from a total of 1913 health check subjects in our hospital from January 2021 to October 2021 were consecutively collected. The inclusion criteria were as follows: (1) age 18 years or older and (2) tube voltage of 120 kVp [7] and availability of QCT bone mineral density measurements. The exclusion criteria [18] were as follows: (1) a field of view (FOV) that did not include the entire lumbar 2 vertebral body; (2) implants, hardware, devices, or other foreign material in lumbar 1 or lumbar 2 vertebra, and (3) Patients with severe degenerative changes or fracture deformity.

**Fig. 1:**
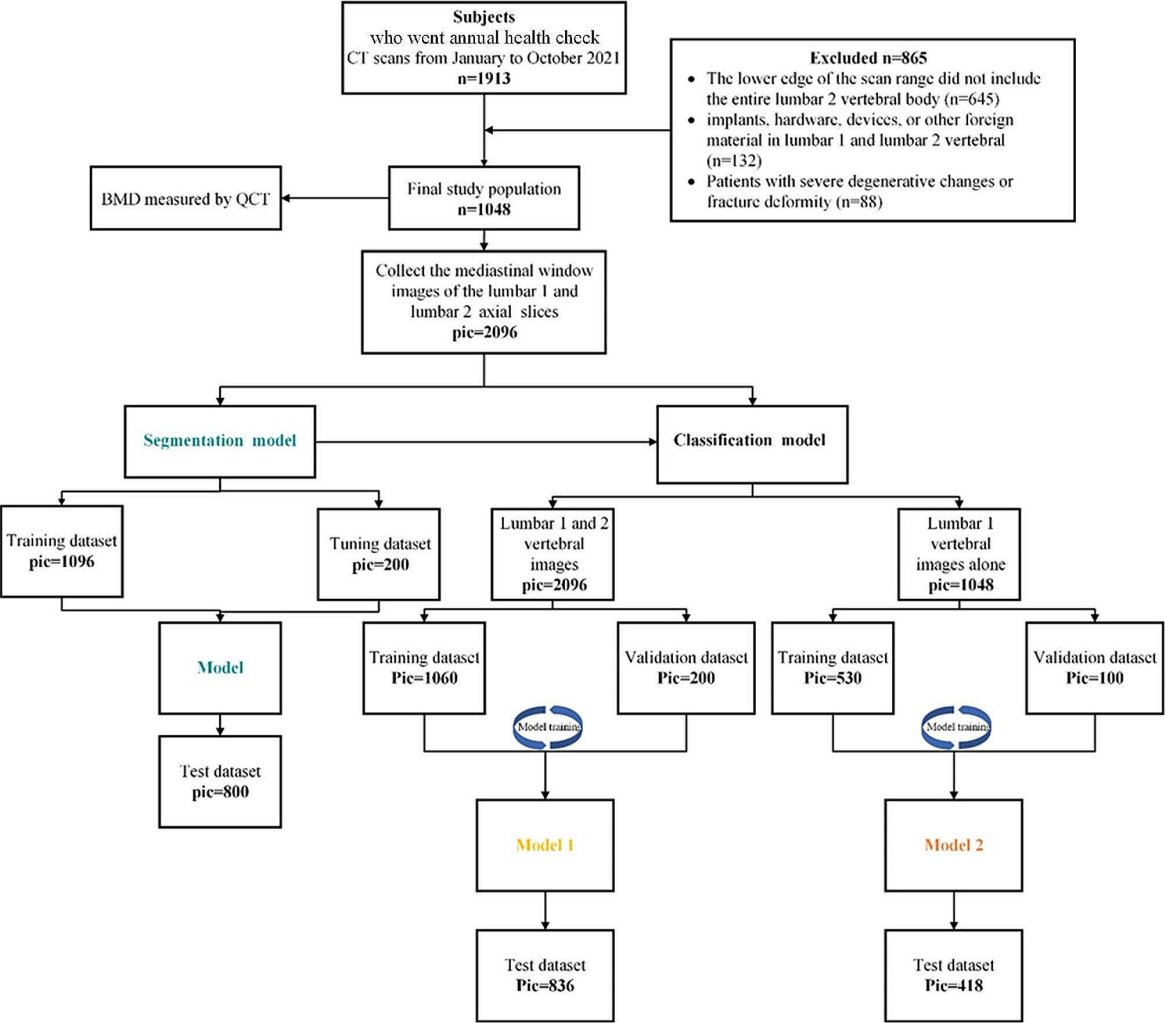
Flowchart for selecting the study population

A total of 1048 subjects were included in the dataset with 865 subjects ruled out. A pipeline depicting patient selection is displayed in Fig. 1.

### CT image acquisition

BMD measurement and model construction were performed on the mediastinal window images of the centre levels of the lumbar 1(L1) and lumbar 2(L2) vertebrae of each subject. We used 2D axial CT slices with a layer thickness of 2 mm.

### Bone mineral density measurement

QCT pro4 software (Mindways, CA, USA) was used to set similarly sized ROIs in the central cancellous bone region of lumbar 1 and lumbar 2, avoiding the cortical bone and the visible blood vessel area. The software automatically calculated the BMD values of the lumbar 1 and lumbar 2 vertebra and used their mean values as the BMD values of the individual subjects (BMD _individual_). According to the criteria recommended by the guidelines [7], BMD _individual_ > 120 mg/cm^3^ was considered normal, 80 mg/cm^3^ ≤ BMD _individual_ ≤ 120 mg/cm^3^ was considered osteopenia, and BMD _individual_ < 80 mg/cm^3^ was considered osteoporosis.

## Model construction

All deep convolution models were completed by Python 3.6.12 software based on the PyTorch framework. The labels of the segmentation model are annotated using Qupath software (https://www.nature.com/articles/s41598-017-17204-5). All experiments were conducted under an Ubuntu 20.04.02 operating system with an NVIDIA GeForce RTX 3090 GPU and 128 GB RAM.

In the data set partitioning section, due to the imbalance of categories in the data set (among the 1048 subjects, the number of individuals with normal BMD status, osteopenia, and osteoporosis were 621, 296, and 131, respectively, and compared to subjects with normal bone mass, the number of subjects with osteopenia and osteoporosis was relatively small), we tried to divide the data set using the traditional 7:1:2 method, but the result is not satisfactory, which affects the confidence of the test results. Therefore, we chose a more balanced dataset partitioning ratio to improve the stability and reliability of the results (the ratio is 5:1:4).

### Image segmentation module

**Fig. 2:**
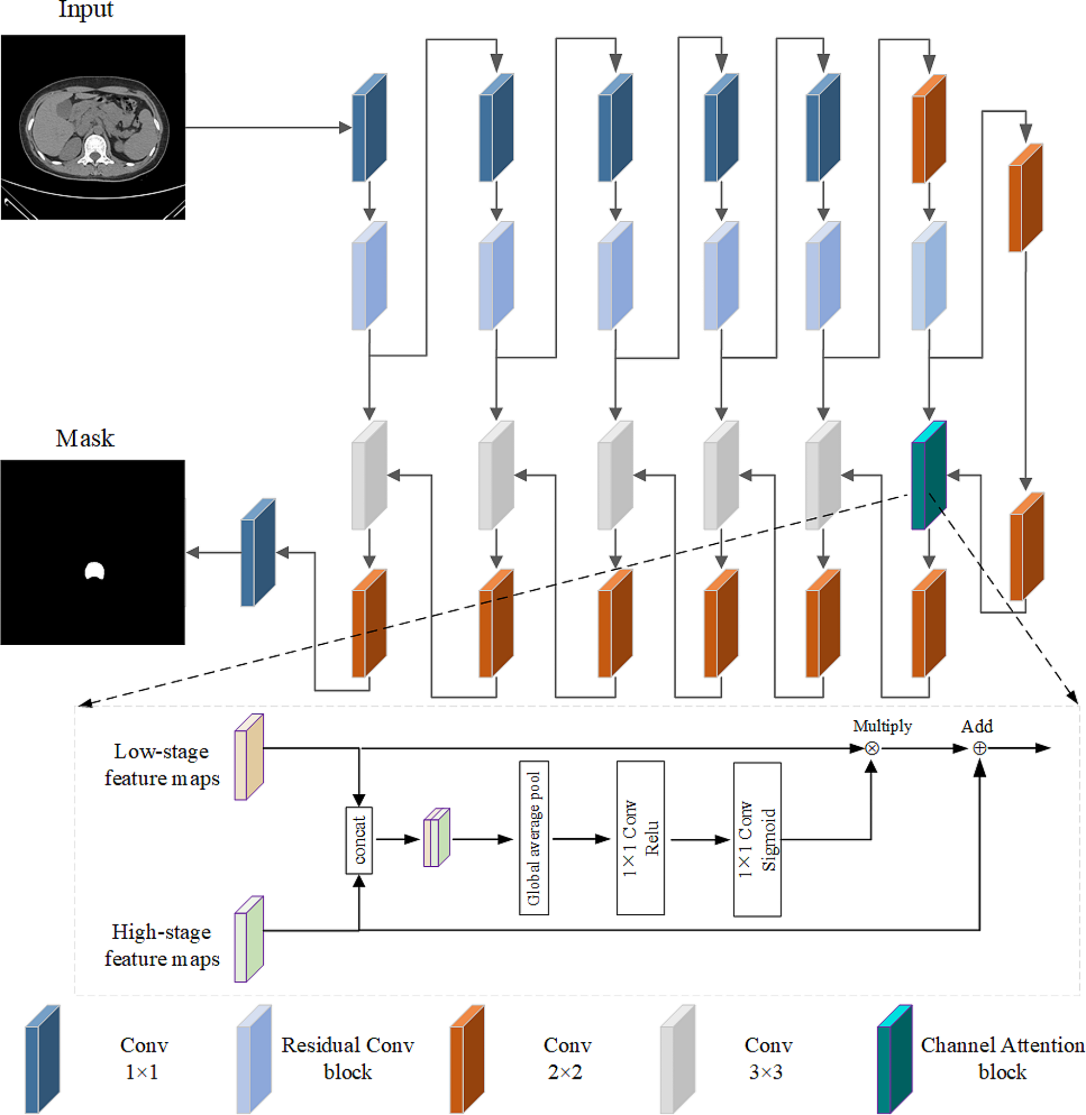
DCNN segmentation structure diagram

First, a deep convolutional neural network (DCNN) segmentation model (Fig. 2) was constructed to automatically segment the vertebral bodies in CT images for subsequent classification. The model adopts a coding-decoding architecture, with a total of seven layers. By adding network layers, the model can learn richer and higher-level feature representations, which helps to improve the model’s understanding and representation of input data. In the first five layers of the coding layer, the convolution of 1 × 1 is first used to increase the nonlinearity. Although the convolution of 1 × 1 has a small sensitivity field in space, it can introduce nonlinearity and increase the expressiveness of the model by combining and interacting the features between channels. Then 3 × 3 convolutional residual structure is used to improve the training depth of the network and enhance the feature transmission. In the sixth layer, 2 × 2 convolution is used instead of 1 × 1 convolution to add receptive fields, which are used to capture local features and structures, such as textures, edges, etc. Only 2 × 2 convolution is used in the seventh layer to reduce resolution and extract higher level features. In the decoding layer, the lowest layer of the decoding layer first uses 2 × 2 transposed convolution to expand the low-resolution feature map, and then adds the channel attention mechanism in the sixth layer to selectively fuse the high-level features of the jump connection layer with the underlying features of the expanded feature map. First, fusion is performed at the channel level through Concat. Then, the feature map dimension is reduced by global average pooling, and the local edge information is highlighted by two 1 × 1 convolution layers. Finally, the weight of each channel obtained is multiplied with the lower-level feature map to obtain the attention vector, and the weight is added with the higher-level feature map to obtain the attention feature map, thus further improving the model segmentation performance. Then 2 × 2 transposed convolution is used to expand the low-resolution feature map, the expanded feature map and the feature map obtained from the skip connection layer are combined by Concat, the local structure and features of the image are extracted by 3 × 3 convolution, and the low-resolution feature map is expanded by 2 × 2 transposed convolution, and so on to the top level. Finally, the convolution of 1 × 1 is used to change the dimension of the model to match the number of model output channels. Here we add a comparison with the segmentation effect of the classical u-net model. First, the depth of the model is improved, which means that there is better nonlinear representation, and more complex variants can be learned to fit more complex feature inputs. Secondly, the channel attention mechanism is introduced to calculate the importance of each channel in the input image through the network, improve the feature representation ability, and be more accurate in the classification area. In addition, the model will obtain higher accuracy and higher AUC values.

The 2096 images were randomly divided into a training set (*n* = 1096), tuning set (*n* = 200) and test set (*n* = 800) at a 5:1:4 ratio.The input data were 512 × 512 images and annotations, and the output data were the results predicted by the model. Image augmentation methods included random flipping, random rotation, left and right mirroring, and random cropping. An experienced radiologist with 3 years of experience in musculoskeletal imaging diagnosis manually drew the ROIs along the inner edge of the cortical bone at the central level of the lumbar 1 and lumbar 2 vertebra with Qupath software to extract lumbar vertebral images. The dice similarity coefficient (DSC) was automatically calculated by the software to compare the consistency between the automatic segmentation of the DCNN and manual labelling. The number of training rounds was 500, and the batch size was 64. The stochastic gradient descent(SGD) optimizer was selected as the optimizer, and the learning rate parameter was 0.001.

### DCNN classification model construction

**Fig. 3:**
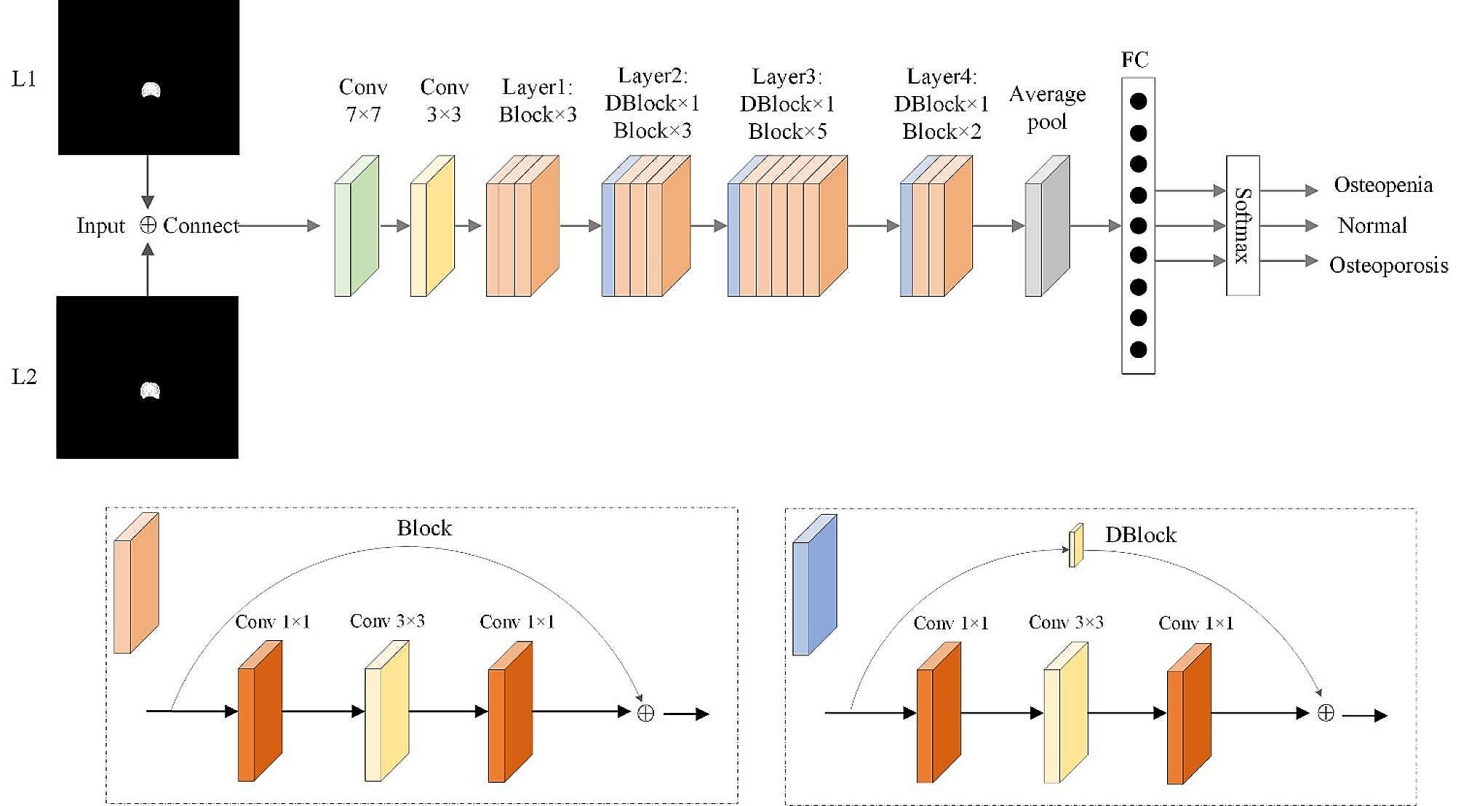
Workflow of the classification model

ResNet-101 residual DCNN classification model (Fig. 3) for input segmentation ROI (pixel size 512 × 512) as model input. The initial layer of the model consists of one 7 × 7 convolutional layer responsible for extracting basic features from the input image. Subsequently, downsampling is performed through one 3 × 3 max-pooling layer to reduce the image size. Following this, there are four residual block layers. The first residual block layer comprises three Block residual units. Each Block residual unit consists of two 3 × 3 convolutional layers for downsampling, reducing the image size, one 1 × 1 convolutional layer to introduce non-linearity, and a residual connection. The residual connection facilitates stable gradient propagation, addressing the issue of gradient disappearance. The second residual block layer includes one DBlock residual unit and three Block residual units. The DBlock differs in that its convolutional operations in the skip structure can transform or dimensionally reduce the features in the skip connection. This ensures that the features in the skip connection have the same dimensions as the subsequent layers, enabling them to be added or concatenated together. The third residual block layer consists of one DBlock residual unit and five Block residual units. The fourth residual block layer comprises one DBlock residual unit and two Block residual units. The purpose of these residual block layers is to learn hierarchical feature representations in the image. Following the output of the last residual block, global average pooling is applied to convert the entire feature map into a vector, capturing global image information. The final fully connected layer is utilized to map high-level features to the number of categories relevant to the osteoporosis classification task, resulting in a three-class deep learning model.

Model 1 was constructed by using the lumbar 1 + lumbar 2 vertebral bodies fusion feature images (by adding feature channels) through the above process. Lumbar 1 image alone were used to construct model 2. The main dataset was constructed from the CT images of 630 subjects from January 2021 to July 2021 (randomly divided into a training dataset and validation dataset with a ratio of 5:1). The remaining 418 patients comprised the test dataset.

### Statistical analyses

All statistical analyses were performed using Python 3.6.12 and SPSS 27. The receiver operating characteristic (ROC) curve was used to analyse the evaluation efficacy of each model on bone mass. The AUC and 95% confidence interval (CI), sensitivity (Se), specificity (Sp), positive predictive value (PPV), negative predictive value (NPV) and accuracy (Ac) were calculated from ROC analysis. The Delong test was used to compare the difference in bone mass evaluation efficiency for the test sets. Numerical data are expressed as the mean ± standard deviation ($\stackrel{-}{x}$±s), and comparisons between groups were performed with variance analysis or the t test. Classification data are expressed as the frequency and percentage (n, %), and the chi-square test was used for comparisons between groups. *P* < 0.05 was considered statistically significant.

## Results

### Baseline and clinical characteristics of the collected data

Among 1048 participants (mean age 51 ± 14.5 years, range 20–92 years), 605 participants were male, and 443 participants were female. There were 621 patients with normal BMD status (the mean BMD was 168.33 ± 23.12 mg/cm^3^ and ranged from 120.30 mg/cm^3^ to 295.10 mg/cm^3^), 296 with osteopenia (the mean BMD was 99.35 ± 10.91 mg/cm^3^, ranging from 80.00 mg/cm^3^ to 120.00 mg/cm^3^) and 131 with osteoporosis (the mean BMD was 60.32 ± 16.48 mg/cm^3^ and ranged from 5.70 mg/cm^3^ to 79.90 mg/cm^3^).

**Fig. 4:**
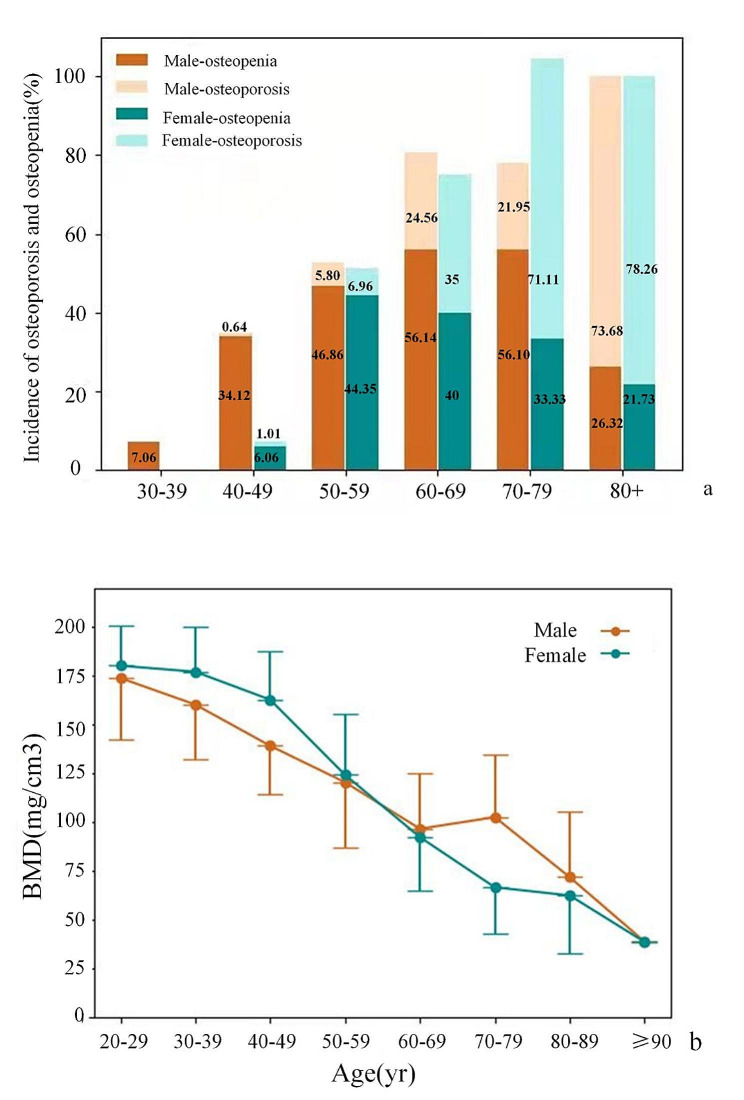
BMD distribution of subjects of all ages. **a** Osteopenia and osteoporosis incidence histogram of different sexes and ages. **b** Different sex and age groups of bone mineral density mean and standard deviation distribution

The distribution of BMD in all age groups is shown in Table 1; Fig. 4. There were significant differences in gender distribution among different age groups (χ^2^ = 22.91, *P* = 0.002). There were significant differences in the average BMD of different age groups and BMD of males and females (*F* = 157.79, 54.85, 141.94, respectively, *P* < 0.05).

**Table 1:** Distribution of BMD in different age groups

Age(yr)	Data set			BMD _individual_ (mg/cm^3^)				*t*	*P*
	Overal	Male	Female		Average	Male	Female		
< 30	60	40	20	175.96 ± 28.27		173.85 ± 31.38	180.18 ± 20.80	0.814	0.419
30 ~ 39	165	85	80	168.42 ± 28.26		160.27 ± 28.37	177.08 ± 23.22	4.174	< 0.001
40 ~ 49	256	157	99	148.27 ± 27.58		139.14 ± 25.06	162.76 ± 25.18	6.899	< 0.001
50 ~ 59	322	207	115	121.83 ± 32.53		120.48 ± 33.20	124.25 ± 31.30	0.470	0.639
60 ~ 69	117	57	60	94.46 ± 28.42		96.66 ± 29.52	92.36 ± 27.41	-0.704	0.483
70 ~ 79	86	41	45	83.83 ± 33.16		102.64 ± 31.91	66.7 ± 23.85	-6.141	< 0.001
80 ~ 89	40	17	23	66.49 ± 31.20		71.88 ± 33.45	62.51 ± 29.54	-1.018	0.315
≥ 90	2	1	1	38.78 ± 20.80		38.85 ± 0.00	38.7 ± 0.00		
*F*		22.91*		157.79		54.85	141.94		
*P*		0.002		0.002		< 0.001	0.038		

*Note* BMD-Bone mineral density. *F*-Variance analysis of bone mineral density differences in subjects of different ages. *-chi-square test. *t*-test of bone mineral density difference between different sexes in the same age group

Figure 4a shows the incidence of osteopenia and osteoporosis in men and women aged 30 years and above in each decade of age. The incidence of osteoporosis increased with age. The incidence of osteoporosis in males aged 50–59, 60–69, 70–79 and over 80 years old was 5.80%, 24.56%, 21.95% and 73.68%, respectively. The incidence of osteoporosis in women of the same age group was 6.96%, 35.00%, 71.11% and 78.26%, respectively. Compared with osteoporosis, osteopenia appeared earlier. The incidence of osteopenia was 34.12% in men and 6.06% in women aged 40 to 49 years. 46.86% and 44.35% in the 50- to 59-year-old group, respectively. The BMD of the lumbar spine in women was higher than that in men (*P* < 0.010) and then gradually decreased with increasing age. The BMD gradually decreased from 162.76 mg/cm^3^ to 38.7 mg/cm^3^. Women had lower BMD than men after 70 years of age (BMD_male_=92.18 ± 34.54mg/cm^3^, BMD_female_=65.07 ± 25.43mg/cm^3^, t = 5.916, *P* < 0.001).

The distribution of subjects and baseline characteristics of each dataset are shown in Table 2. There was no significant difference in the sex distribution of the subjects in each dataset (*x*^2^ = 0.862, *P* = 0.650). There was no significant difference between age and BMD (*F* = 0.255, 0.084, *P* = 0.775, 0.919). The ratio of normal, osteopenia and osteoporosis cases was close to 6:3:1.

**Table 2:** Subject distribution and baseline characteristics of each dataset

		Overall	Training dataset	Validation dataset	Test dataset	*F*	*P*
		*n* = 1048	*n* = 530	*n* = 100	*n* = 418		
Gender, n, %	Male Female	605, 57.73%	305, 57.55%	62, 62.00%	238, 56.94%	0.862*	0.650
		443, 42.27%	225, 42.45%	38, 38.00%	180, 43.06%		
Age(yr.), mean ± SD		51.19 ± 14.35	51.04 ± 14.4	51.11 ± 12.73	51.70 ± 14.97	0.255	0.775
BMD (mg/cm^3^), mean ± SD		130.28 ± 42.76	129.92 ± 42.78	131.30 ± 39.29	130.90 ± 42.96	0.084	0.919
BMD categories, n (%)	Normal	621, 27.96%	310, 58.49%	60, 60.00%	251, 60.05%		
	Osteopenia	296, 59.64%	150, 28.30%	30, 30.00%	116, 27.75%	0.994*	0.911
	OP	131, 12.40%	70, 13.21%	10, 10.00%	51, 12.20%		

*Note* BMD-Bone mineral density. *F*-Variance analysis of bone mineral density differences in subjects of different ages. *- chi-square test

### The overall diagnostic efficiency of the DCNN model

**Fig. 5:**
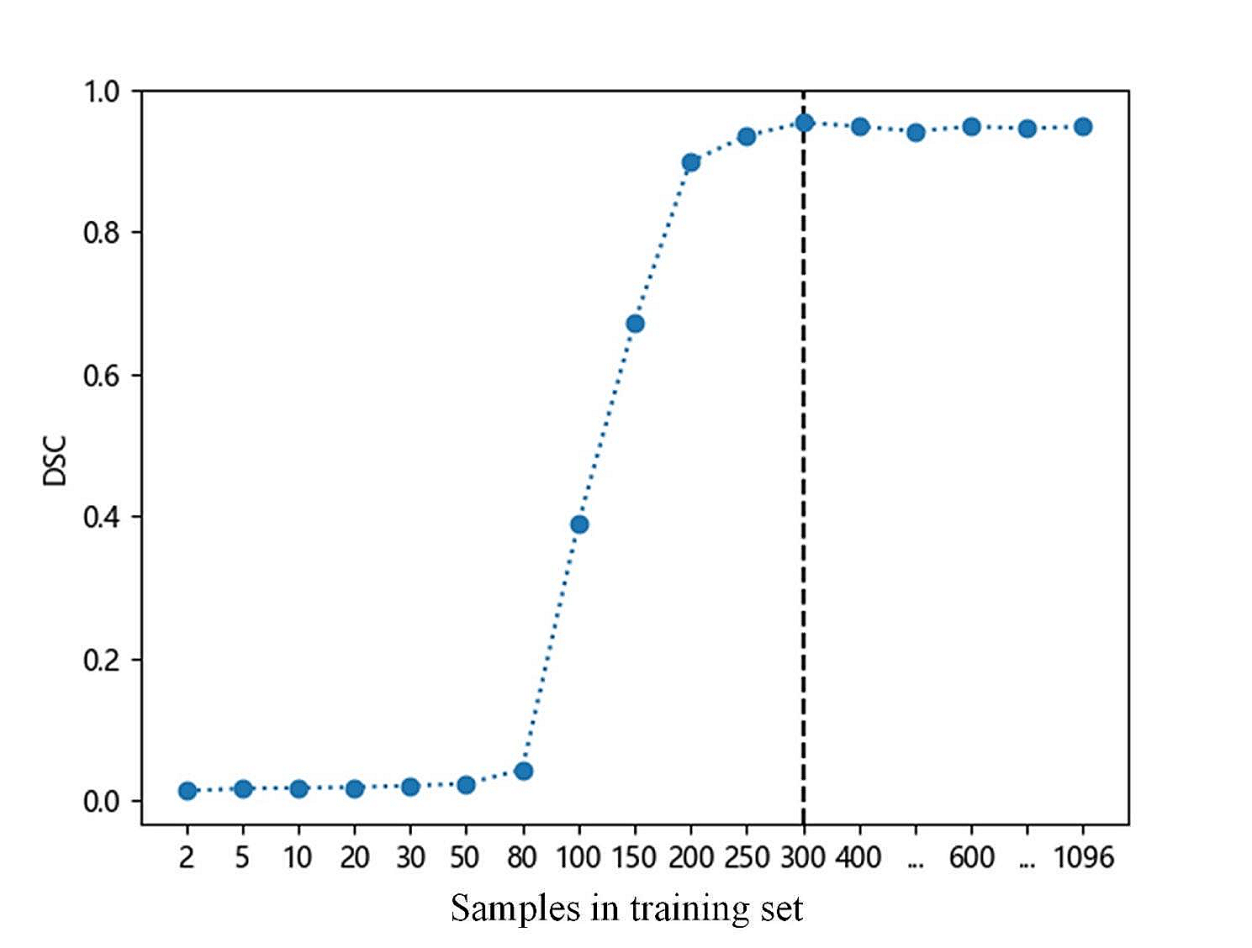
The relation curve between segmentation model performance and the sample numbers of training set

**Fig. 6:**
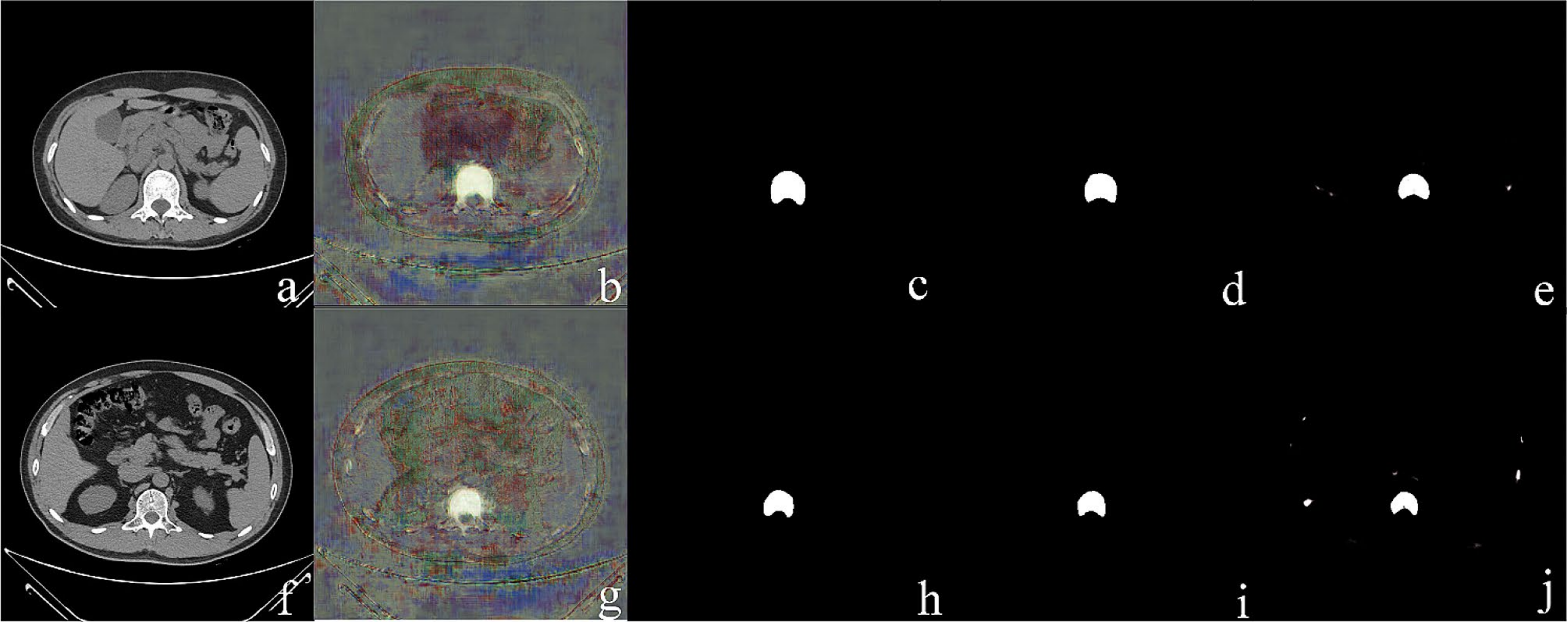
Partial segmentation results. **a-e**: The female was 34 years old, who was diagnosed as normal bone mass with BMD _individual_=196.6 mg/cm3. Fig. f-j: The male was 43 years old, who was diagnosed as osteopenia with BMD _individual_=118.3 mg/cm3. **a**, **e**: Axial CT images of the central slice of the lumbar 1 vertebra. **b**, **g**: Heatmap of predicted probabilities during training. **c**, **h**: manual segmentation label map; **d**, **i**: DCNN segmented image; **e**, **j**: U-net segmented image

**Fig. 7:**
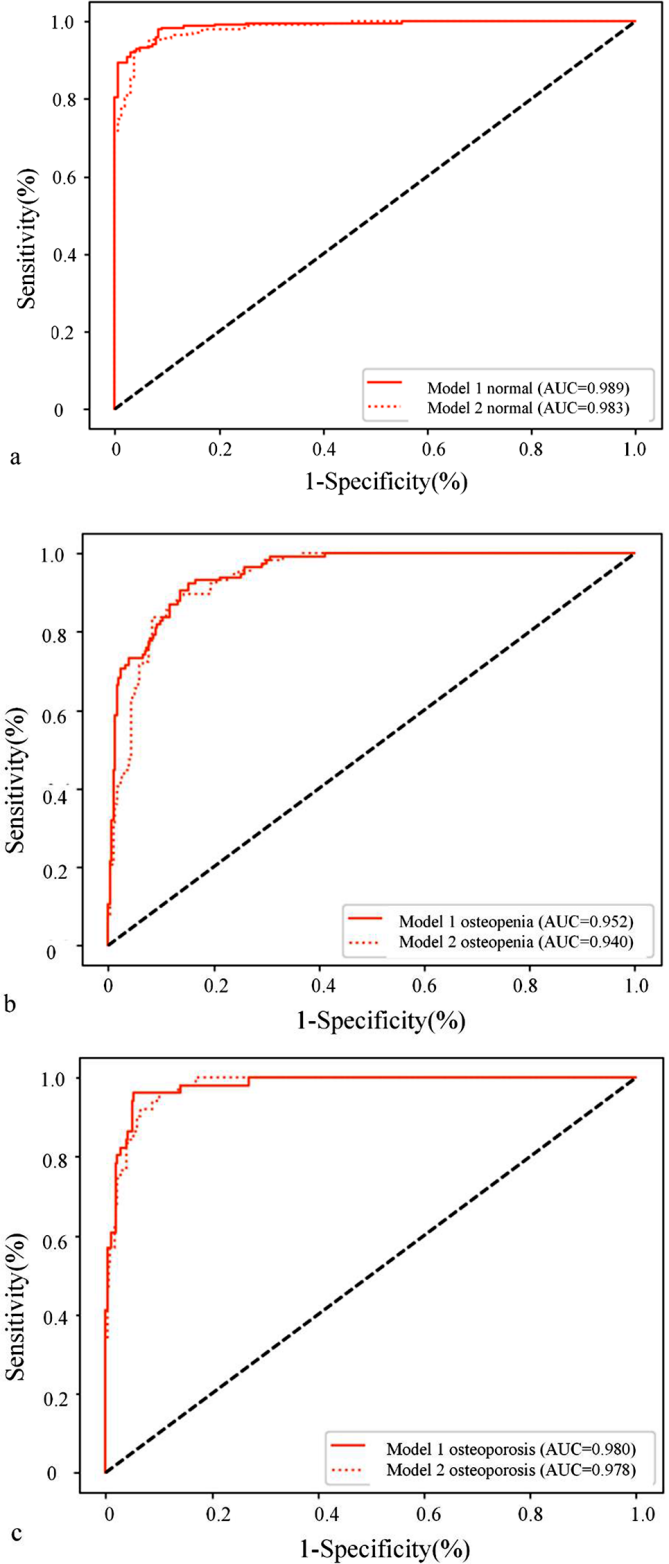
ROC curve for the two models. **a-c**: Comparison of the evaluation efficacy of model 1 and model 2 in the test set for normal, osteopenia, and osteoporosis

As shown in Fig. 5, when the number of images in the training set was greater than 300, the DSC value tended to be stable and when the number of images in the training set was 300–1096, the DSC value was 0.95 ± 0.004. When the number of images in the training set was 300, the DSC value was 0.951 ± 0.03 in the test set. This is sufficient to ensure the performance of vertebral image segmentation. The segmentation effect of some CT images is shown in Fig. 6. The efficacy evaluation of the two models on bone mass in each dataset is shown in Table 3; Fig. 7.

**Table 3:** The performance metrics of the two models

			Model 1			Model 2	
		Training dataset	Validation dataset	Test dataset	Training dataset	Validation dataset	Test dataset
normal	AUC	0.999	0.972	0.989	0.999	0.966	0.983
	(95%CI)	(0.998, 1.000)	(0.939, 1.000)	(0.983, 0.996)	(0.998, 1.000)	(0.924, 1.000)	(0.974, 0.992)
	Se	0.990	0.917	0.964	1.000	1.000	0.976
	Sp	0.972	0.900	0.916	0.927	0.850	0.838
	PPV	0.981	0.932	0.945	0.951	0.909	0.901
	NPV	0.986	0.878	0.944	1.000	1.000	0.959
	Ac	0.983	0.910	0.945	0.969	0.940	0.921
osteopenia	AUC	0.996	0.942	0.952	0.996	0.929	0.940
	(95%CI)	(0.994,0.999)	(0.890,0.994)	(0.932,0.971)	(0.992, 0.999)	(0.971, 0.987)	(0.919, 0.962)
	Se	0.940	0.833	0.716	0.893	0.767	0.638
	Sp	0.984	0.886	0.960	0.995	0.957	0.954
	PPV	0.959	0.758	0.874	0.985	0.885	0.841
	NPV	0.977	0.242	0.898	0.959	0.905	0.873
	Ac	0.972	0.870	0.892	0.967	0.900	0.866
osteoporosis	AUC	0.999	0.989	0.980	1.000	0.981	0.978
	(95%CI)	(0.997,1.000)	(0.972,1.000)	(0.967,0.994)	(0.999, 1.000)	(0.957, 1.000)	(0.965, 0.990)
	Se	0.957	0.700	0.941	0.971	0.700	0.843
	Sp	0.993	0.989	0.948	1.000	0.989	0.959
	PPV	0.957	0.875	0.716	1.000	0.875	0.741
	NPV	0.993	0.967	0.991	0.996	0.967	0.978
	Ac	0.989	0.960	0.947	0.996	0.960	0.945

In the test dataset, the diagnostic efficiency of model 1 for normal was better than that of model 2, and the difference between the two was statistically significant (*P* = 0.033). There was no significant difference between Model 1 and Model 2 in the diagnostic efficacy of osteopenia and osteoporosis (*P* = 0.21 and 0.546, respectively) (Table 4).

**Table 4:** Comparison of the efficacy of different DCNN models for bone mass assessment in the test dataset

	AUC_1_	AUC_2_	*Z*	*P*
normal	0.990	0.983	2.139	0.033
osteopenia	0.952	0.940	1.247	0.21
osteoporosis	0.980	0.978	0.603	0.546

*Note* AUC_1_ Area under the curve for the model 1 test set; AUC_2_ Area under the curve for the model 2 test set

## Discussion

In this study, we used QCT as the classification standard for bone mass assessment and proposed a deep learning model for osteoporosis screening based on chest CT with L1 + L2 vertebral bodies fusion feature images. Ultimately, considering that the CT images are all chest scan images, we further constructed the model 2 based on the L1 alone. The results confirmed that the diagnostic efficiency of model 2 constructed with only L1 alone was inferior to model 1 constructed with two vertebral bodies when the bone mass was normal. Even it was inferior to model 1, model 2 achieves favorable performance. The model 2 diagnosing normal, osteopenia and osteoporosis achieved an AUC of 0.983, 0.940 and 0.978. The benefits of model 2 which based on the lumbar 1 vertebra alone are obvious: firstly, moving up the lower limit of the CT scanning range from the level of the L2 to the level of the L1 shortens the scanning length of the *z*-axis, reduces the patient’s radiation exposure. Although the reduction of radiation dose may be limited for a single individual, considering the huge number of annual health check population, this change is quite significant in terms of reducing the overall radiation dose of the population. Secondly, according to the guidelines for the use of QCT [18], BMD is generally measured using L1 and L2, which requires that the scanning range should include L2, and in general, the lower boundary of both lungs is located at the level of thoracic 12 vertebra [19]. Since there is no need to intentionally expand the scanning range and require additional requirements or training for medical imaging technologists, they can perform chest CT scanning according to usual habits, thereby avoiding additional training costs. Finally, using a single image based DCNN model for opportunistic osteoporosis screening has the potential to reduce the data collection costs and alleviate the storage pressure on the post-processing workstation.

In this study, the prevalence of osteoporosis was 15.4% in men and 32.7% in women over 50 years old. The prevalence of osteopenia was 39% in men and 49% in women over 50 years old. This result is similar to the results of Cheng [2] et al. who evaluated the bone mass distribution of Chinese men and women by QCT in a large sample study. In the study by Cheng et al., the prevalence of osteoporosis in Chinese men and women over 50 years old was approximately 14% and 29%, respectively, and the prevalence of osteopenia was 42% for both. The population distribution of this study is consistent with the distribution of populations at high risk of osteopenia and osteoporosis in China currently, making the results of the study more reliable.

At present, DXA is often used as a reference standard for bone density classification in clinic to diagnose osteoporosis. In a study based on lumbar CT images of 808 postmenopausal women, Zhang [14] et al. used DXA as the bone mass classification standard to construct a DCNN model for the diagnosis of osteoporosis, with a sensitivity of 68.4%, specificity of 67.8%, and AUC of 0.726. In this study, QCT was used as the classification standard for bone mass assessment, and the diagnostic performance of the model was better than the results of Zhang et al. DXA is a two-dimensional imaging technique, which is susceptible to scoliosis, facet joint degeneration, soft tissue calcification, especially abdominal aorta calcification and other factors, which reduces the accuracy of bone density measurement. QCT is a three-dimensional imaging technique that can quantitatively evaluate the bone density of the vertebral cancellous bone that is more sensitive to OP, and the measured results are more stable than DXA [8, 9].

This study confirmed the feasibility of opportunistic osteoporosis screening based on chest CT images. Based on U-Net [15], we designed a new segmentation model. First, the model depth improved, meaning a better nonlinear expression ability, and it can learn more complex transformations to fit more complex feature inputs. Second, the channel attention mechanism is introduced to calculate the importance of each channel of the input image through the network to improve the feature representation ability making the regions used for classification more accurate. Additionally, the model will obtain higher accuracy and higher AUC values. Next, we used ResNet-101 residual DCNN classification model to classify the segmentation images, which achieved achieve satisfactory results.

Tang [20] et al. have reported that the opportunistic osteoporosis screening using L1 trabecular attenuation with an accuracy of 76.65% and an AUC of 0.917. Compared with the data, our results are higher than the results in that paper. The reason may be that they used 2D axial CT slices with a layer thickness of 5 mm, while we used original axial CT slices, and the classification model we used was ResNet. Compared with the DenseNet convolutional neural network model with dense connections, the ResNet model introduces a residual network structure, which can effectively avoid the gradient disappearance and gradient explosion problems with increasing depth in network training so that the model can be better fitted. The ResNet-101 residual DCNN model may be more suitable for screening opportunistic osteoporosis or osteopenia in a large-scale annual health check population and provide the possibility for early clinical diagnosis and intervention.

We only used 2D axial CT slices with a layer thickness of 2 mm to initially explore whether 2D CT slices can provide relevant reference value. From the perspective of research design, many previous studies [21, 22] evaluated sagittal images. Leonhardt Y [21] et al. used 58 patients’ sagittal CT scans to assess whether BMD measured with QCT can predict osteoporotic fracture occurrence in a prospective clinical cohort, ROC showed an AUC of 0.76 and a Youden’s Index of J = 0.48. Lee SJ [22] et al. used 571 consecutive adults’ sagittal reconstruction abdominal CT scans obtained for other purposes for vertebral fracture assessment. The difference lies in the fact that we use the axial original images, which does not need to be reconstructed. When the sample size is large, it saves time and storage space compared with the reconstructed sagittal images. And, there may be imprecise for not using original images and it’s not practical in clinics using sagittal images.

Although the proposed method achieved convincing results, some limitations should be mentioned. First, this was a single-center retrospective study, and external validation was limited by hardware constraints. Second, in this study, the DCNN model was constructed only for 2D axial CT images with a slice thickness of 2 mm, and all 3D image features of the target vertebral body were not included. However, the DCNN model has the advantages of a simple structure, small data demand, low computational complexity, and short training time. Moreover, the annotation of segmentation was provided by one radiologist with 3 years of experience, which might be subjected to observer variability. Since the cases were collected consecutively, the sample contain fewer female participants than male. The individual anatomical variation should be considered as a potential element that affecting the selection of the optimal site, it is evitable to some extent. Collecting more data for training may enhance the robustness. Further study is needed to validate our findings in multi-center data. Finally, the model construction in this study was based only on CT image features, and other clinical information, such as age, sex, and body mass index, were not considered. The above shortcomings need to be considered in subsequent clinical research and application.

In conclusion, this study proposed a deep learning model based on chest CT that achieves favorable performance on opportunistic osteoporosis screening using the lumbar 1 + lumbar 2 vertebral bodies fusion feature images, then further constructed the comparable model based on the lumbar 1 vertebra alone. The method can shorten the scan length, reduce the radiation dose received by patients, and reduce the training cost of technologists. Our method may improve the diagnosis of osteoporosis and help health check subjects to prevent bone mass loss. The establishment of this model can help clinical screening of large sample population, find out the cases that really need clinical intervention, and can also be used for diagnosis and treatment evaluation and follow-up work.

## Data Availability

The datasets generated and/or analysed during the current study are not publicly available due the clinical parameters of the patients involved in this paper were owned by the First People’s Hospital of Nantong, China, the hospital have agreed to participate in the survey on condition that the collected data may not passed on to any third parties. Or more precisely, data are not publicly accessible and freely available. but are available from the corresponding author on reasonable request.

## Abbreviations

CT: Computed tomography
DXA: Dual-energy X-ray absorptiometry
BMD: Bone mineral density
AUC: Area under the curve
CNN: Convolutional neural network
WHO: World Health Organization
ROI: Region of interest
CI: Confidence interval
QCT: Quantitative computed tomography
FOV: Field of view
SCOOP: Screening for prevention of fractures in older women
L1: Lumbar 1 vertebra
L2: Lumbar 2 vertebra
SGD: Stochastic gradient descent
